# Between compulsions and contagions: examining the protective role of OCD against COVID-19 in a large cohort study

**DOI:** 10.3389/fpsyt.2024.1464353

**Published:** 2024-11-25

**Authors:** Chen Avni, Dana Sinai, Paz Toren

**Affiliations:** ^1^ Ramat-Chen Brüll Mental Health Center, Clalit Health Services Community Division, Tel-Aviv, Israel; ^2^ Faculty of Medical & Health Sciences, Tel Aviv University, Tel-Aviv, Israel; ^3^ Baruch Ivcher School of Psychology, Reichman University, Herzliya, Israel

**Keywords:** obsessive-compulsive disorder (OCD), COVID-19, infection rates, vaccination rates, psychoeducation

## Abstract

**Introduction:**

Since the onset of the COVID-19 pandemic in 2020, a significant body of research has explored the impact of the virus and its preventative measures on mental health among individuals with OCD. However, to our knowledge, no study has been conducted to test whether the very behaviors considered symptomatic of OCD inadvertently offer a protective shield against COVID-19 infection.

**Methods:**

This retrospective cohort study utilized the electronic health record database of Israel’s largest healthcare provider, Clalit Health Services (CHS), to compare patients with and without recorded OCD diagnoses in terms of the number of COVID-19 tests taken, hospitalizations, vaccination rates, and infection rates during and after different pandemic waves.

**Results:**

The OCD group had a slightly higher rate of positive COVID-19 tests compared to the control group (*p*<0.001), but only since the easing of restrictions after the end of the fifth wave. The OCD group was also more likely to receive a third dose of the COVID-19 vaccine (*p*<0.001).

**Discussion:**

Our findings suggest that OCD may not confer protection against COVID-19 and may even be associated with slightly higher infection rates, particularly in the post-restrictions period.

## 1 Introduction

Obsessive-Compulsive Disorder (OCD) is a chronic mental health condition affecting 1–3% of individuals, characterized by obsessions—unwanted, recurring thoughts—and compulsions—repetitive behaviors performed to alleviate distress. Symptoms often persist and can worsen over time, leading to significant impairment and reduced quality of life. The etiology of OCD is multifactorial, involving genetic, neurobiological, and environmental factors (1, 2).

In patients with OCD, the relentless dread of contamination is the most commonly observed symptom (3, 4). Contamination fear is the fear of direct or indirect contact with a person or item perceived as dirty or harmful (5). This form of OCD is marked by enduring and overarching concerns about contamination, a characteristic widely recognized as a defining, distinctive, and predominant facet of the disorder (6). Individuals with these obsessions often engage in compensatory behaviors such as excessive washing, cleaning, and checking to eliminate the perceived threat of contamination and protect themselves from various feared outcomes, such as illness. This aspect of OCD can dominate a person’s life, leading to significant distress and impairment. Moreover, the fear of contamination can lead to a preoccupation with not only personal cleanliness but also with the cleanliness of one’s surroundings, prompting avoidance of anything considered contaminant (1).

From an evolutionary standpoint, some OCD characteristics, especially those related to cleanliness and avoiding germs, might have offered survival benefits in the past. Behaviors aimed at reducing exposure to pathogens could have been beneficial in ancestral environments where infectious diseases were a major cause of mortality. This perspective suggests that certain OCD traits may be an exaggeration of evolutionarily adaptive behaviors evolved to avoid harm, with the emergence of a fully-fledged disorder in a minority of the population representing a pathological extreme of these otherwise advantageous traits (7, 8).

In early 2020, Coronavirus disease 2019 (COVID-19), caused by the novel coronavirus SARS-CoV-2, emerged as a global pandemic. It has posed unprecedented challenges to public health, economies, and social structures worldwide. The virus primarily spreads through respiratory droplets, necessitating public health measures such as social distancing, mask-wearing, and enhanced hygiene practices (9).

The COVID-19 pandemic has brought unique considerations for individuals with OCD, especially those with contamination fears. However, studies have shown that OCD symptoms did not universally exacerbate during the COVID-19 pandemic as previously feared (10–12). This period has also sparked renewed interest in the idea that certain obsessive-compulsive behaviors may offer an evolutionary advantage in reducing infectious disease transmission. The emergence of COVID-19 provided a critical moment to explore this concept further (13).

While there is an increasing amount of research on how COVID-19 and its prevention strategies have affected the mental health and symptoms of those with OCD (14), there remains a notable gap in understanding how having OCD affects the likelihood of contracting COVID-19, which this study intends to address.

Existing literature has established that specific comorbidities commonly associated with OCD, such as Attention-Deficit Hyperactivity Disorder (ADHD) and Schizophrenia (15–18), may affect one’s chances of contracting COVID-19. However, to our knowledge, no study has specifically examined the direct impact of OCD diagnosis on COVID-19 infection rates.

Using the database of Clalit Health Services (CHS), the largest healthcare provider in Israel, this study examines whether OCD may provide any protective benefits against COVID-19 and explores the complex interactions between OCD and susceptibility to the virus.

## 2 Materials and methods

The study was approved by the institutional review boards (Study designation 0143-22-COM). It was conducted in accordance with the International Conference on Harmonisation guidelines and ethical principles of the Declaration of Helsinki.

### 2.1 Study design and procedure

This retrospective cohort study was conducted using CHS’s electronic health record database. CHS is an Israeli payer-provider integrated health care system serving over 4.5 million members, constituting 54% of the Israeli population. The database includes patient demographic and clinical characteristics, hospital discharge and outpatient clinic diagnoses, laboratory test results, medical treatments, and medication dispensation information. Data was accessed and extracted from the CHS database using the Clalit Research Data secure anonymized data-sharing platform powered by MDClone (https://www.mdclone.com).

The dataset was used to investigate the differences in COVID-19 infection rates between patients with and without a recorded diagnosis of OCD.

To delineate the progression of the COVID-19 pandemic, distinct waves were defined based on COVID-19 wave data collected and published by the Israeli Ministry of Health (19). The first COVID-19 wave spanned from March 1^st^ to May 31^st^, 2020, followed by the second wave from June 1^st^ to October 30^th^, 2020. The third wave was from November 1^st^, 2020, to April 30^th^, 2021, the fourth from May 1^st^ to October 30^th^, 2021, and the fifth wave extended from November 1^st^, 2021, to April 30^th^, 2022. Mask restrictions were lifted on all low-risk settings in Israel on April 23^rd^ 2022 (20).

### 2.2 Inclusion criteria

Patients were eligible if they were born on or before February 1^st^, 2016, ensuring they were at least four years old at the onset of the pandemic. Additionally, they needed to have been insured by CHS on or before February 1st, 2020. The coverage by CHS needed to extend up to February 1^st^, 2023, or until the patient’s demise, whichever occurred first. These criteria ensured a consistent and comprehensive patient data analysis over the pandemic’s specified duration.

### 2.3 Measures

Demographic information, including gender and socioeconomic status (SES) categorized as Low, Medium, or High, was obtained from CHS’s computerized database. Age was computed based on the year of birth, establishing the age of participants as of the year 2020.

OCD diagnosis: Patients were classified as having an OCD diagnosis based on the presence of any diagnosis under the ICD-10 code F42.

Comorbid psychopathology: This study assessed comorbid psychopathology by identifying psychiatric conditions through historical ICD-10 diagnoses, including: Post-traumatic stress disorder (PTSD) as F43.1; Attention-deficit hyperactivity disorder (ADHD) as F90; Schizophrenia as F20; Schizoaffective disorder as F25; and Bipolar disorder as F31, excluding cases with schizoaffective disorder or schizophrenia diagnoses. Anxiety was categorized under F40.0, F40.2, F41.0, F41.1, and F41.9, while Depression was identified through F32 and F33, omitting cases with bipolar, schizoaffective, or schizophrenia disorders. Panic and agoraphobia were specified with codes F41.0 and F40.01, respectively.

Severe psychopathology was defined as a historical diagnosis of either Schizophrenia or schizoaffective disorders, and Any psychopathology was determined by the presence of any psychiatric diagnosis.

### 2.4 Statistical analysis

Initially, we compared the entire sample, which included the control group, with the OCD group in relation to sociodemographic characteristics and the existence of any psychiatric disorders. Categorical variables were assessed using the χ2 test, while continuous variables were examined utilizing an independent-samples t-test.

After a preliminary review, we matched individuals with OCD to controls, ensuring a balanced comparison across sociodemographic and comorbid psychopathology variables. Matching was conducted on age, gender, socioeconomic status, severe psychopathology, any psychopathology, ADHD diagnosis, and bipolar disorder diagnosis. Leveraging the control group’s large size relative to the OCD group we attained a precise 1:1 match for all variables, resulting in identical distributions between the OCD and control groups. When multiple controls fit an OCD case, we randomly selected one to pair with each OCD individual.

To compare the OCD and control groups across COVID-19 outcomes, we performed logistic regression analyses for binary outcomes (e.g., positive COVID-19 test, wave-specific positivity, hospitalization, and vaccination) and linear regression analyses for continuous outcomes (e.g., number of COVID tests). For each binary outcome, two logistic regression models were fitted. The first model was unadjusted, predicting the outcome from the group (OCD vs. control). The second model adjusted for psychiatric comorbidities that differed between the groups, specifically PTSD, schizoaffective disorder, panic disorder, anxiety, and depression. Odds ratios (ORs) and 95% confidence intervals (CIs) were calculated for each model. For the continuous outcomes, linear regression models were used to estimate unadjusted and adjusted differences in means (β coefficients) between the OCD and control groups, with 95% CIs reported. Additionally, chi-square tests were used to compare the proportions between the OCD and control groups for each binary outcome, with the corresponding p-values reported.

We conducted a Kaplan-Meier survival analysis to evaluate the time to the first positive COVID-19 test for individuals with OCD compared to the control group. Additionally, we performed Cox proportional hazards regression models to analyze the effect of OCD on the time to first positive COVID-19 test, using hazard ratios (HRs) to assess the risk. Both unadjusted and adjusted models were fitted. The adjusted model controlled for psychiatric comorbidities, including PTSD, schizoaffective disorder, panic disorder, anxiety, and depression.

The subsequent analysis compared matched OCD individuals and controls, examining COVID-19 infection rates, testing frequency, hospital stays, and vaccination rates. A linear regression predicted the time to the first positive result for those testing positive, considering the same variables. Additionally, a Cox proportional hazards regression analyzed OCD’s impact on infection timing, using hazard ratios to assess risk.

All statistical analyses were conducted using R version 4.2.3.

## 3 Results

### 3.1 Descriptive statistics and baseline group comparison

The sample included N=3,281,540 members in the control group and N=15,436 in the OCD group. **Table 1** shows baseline differences: the OCD group was younger (mean age 35.62 vs. 39.68; *p*<0.001) and had more males (52.3% vs. 47.1%; *p*<0.001). Socioeconomic status varied, with OCD participants represented more in the highest and lowest brackets (18.5% vs. 16.6% and 59.2% vs. 52.8%; *p*<0.001). Severe psychiatric comorbidities were more common in the OCD group, with 15.1% showing severe psychopathology versus 1.3% in controls and 68.0% having any psychopathology compared to 19.6%. ADHD prevalence was also higher in the OCD group (21.4% vs. 6.1%).

**Table 1:** Sample characteristics.

Characteristic	Control Group (N=3,281,540)	OCD Group (N=15,436)	Statistic (t/χ²)	*p-value*
Age, mean (SD)	39.68 (24.88)	35.62 (19.82)	t = 25.40	<0.001*
Gender, n (%)			χ² = 161.93	<0.001*
Male	1,546,893 (47.1%)	8,068 (52.3%)		
Female	1,734,645 (52.9%)	7,368 (47.7%)		
Socioeconomic status, n (%)			χ² = 512.79	<0.001*
High	544,350 (16.6%)	2,852 (18.5%)		
Medium	794,500 (24.2%)	2,607 (16.9%)		
Low	1,732,479 (52.8%)	9,142 (59.2%)		
No data	210,211 (6.4%)	835 (5.4%)		
Severe Psychopathology, n (%)	41,337 (1.3%)	2,336 (15.1%)	χ² = 22613.93	<0.001*
Any Psychopathology, n (%)	641,859 (19.6%)	10,492 (68.0%)	χ² = 22683.90	<0.001*
ADHD Diagnosis, n (%)	201,759 (6.1%)	3,308 (21.4%)	χ² = 6148.77	<0.001*
Bipolar Diagnosis, n (%)	210211 (6.4)	835 (5.4)	χ² = 4338.87	<0.001*
PTSD Diagnosis, n (%)	41337 (1.3)	2336 (15.1)	χ² = 3034.81	<0.001*
Schizophrenia Diagnosis, n (%)	641859 (19.6)	10492 (68.0)	χ² = 21373.35	<0.001*
Schizoaffective Diagnosis, n (%)	201759 (6.1)	3308 (21.4)	χ² = 10492.13	<0.001*
Panic Disorder Diagnosis, n (%)	12708 (0.4)	580 (3.8)	χ² = 4443.42	<0.001*
Anxiety Diagnosis, n (%)	43153 (1.3)	992 (6.4)	χ² = 24915.79	<0.001*
Depression Diagnosis, n (%)	39725 (1.2)	2228 (14.4)	χ² = 8259.62	<0.001*

Characteristics of unmatched sample. Severe Psychopathology, Schizophrenia, Schizoaffective disorder; ADHD, Attention Deficit and Hyperactivity Disorder. * *p <*0.001.

### 3.2 Baseline characteristics of matched cohort

After performing exact matching on age, gender, SES, severe psychopathology, any psychopathology, ADHD diagnosis, and bipolar disorder diagnosis, the matched cohort consisted of 15,360 individuals in each group (OCD and control). The characteristics of the matched cohort are shown in **Table 2**.

**Table 2:** Characteristics of matched sample.

Characteristic	Total Sample (n = 30,720)	Control Group (n = 15,360)	OCD Group (n = 15,360)	χ^2^	*p*-value	SMDcenter
Age, mean (SD)	35.63 (19.83)	35.63 (19.83)	35.63 (19.83)		NA	<0.001
Gender, n (%)					NA	<0.001
Female	14662 (47.7%)	7331 (47.7%)	7331 (47.7%)			
Male	16058 (52.3%)	8029 (52.3%)	8029 (52.3%)			
Socioeconomic status, n (%)					NA	<0.001
High	5654 (18.4%)	2827 (18.4%)	2827 (18.4%)			
Medium	18262 (59.4%)	9131 (59.4%)	9131 (59.4%)			
Low	5172 (16.8%)	2586 (16.8%)	2586 (16.8%)			
No Data	1632 (5.3%)	816 (5.3%)	816 (5.3%)			
Severe Psychopathology, n (%)	4606 (15.0%)	2303 (15.0%)	2303 (15.0%)		NA	<0.001
Any Psychopathology, n (%)	20852 (67.9%)	10426 (67.9%)	10426 (67.9%)		NA	<0.001
ADHD Diagnosis, n (%)	6506 (21.2%)	3253 (21.2%)	3253 (21.2%)		NA	<0.001
Anxiety Diagnosis, n (%)	17305 (56.3%)	8308 (54.1%)	8997 (58.6%)	62.64	<0.001*	0.091
Bipolar Diagnosis, n (%)	1080 (3.5%)	540 (3.5%)	540 (3.5%)		NA	<0.001
Depression Diagnosis, n (%)	9384 (30.5%)	4131 (26.9%)	5253 (34.2%)	192.81	<0.001*	0.159
Panic Disorder Diagnosis, n (%)	1429 (4.7%)	470 (3.1%)	959 (6.2%)	174.78	<0.001*	0.152
PTSD Diagnosis, n (%)	1715 (5.6%)	733 (4.8%)	982 (6.4%)	37.98	<0.001*	0.071
Schizoaffective Diagnosis, n (%)	1878 (6.1%)	874 (5.7%)	1004 (6.5%)	9.44	0.002*	0.035
Schizophrenia Diagnosis, n (%)	4404 (14.3%)	2207 (14.4%)	2197 (14.3%)	0.02	0.884	0.002

Characteristics of the matched sample after performing exact matching on age, gender, socioeconomic status, severe psychopathology, any psychopathology, ADHD diagnosis, and bipolar disorder diagnosis. Due to exact matching, the distributions are identical between groups SMD, Standardized Mean Difference; Severe Psychopathology, Schizophrenia, Schizoaffective disorder; ADHD, Attention Deficit and Hyperactivity Disorder; PTSD, Post-Traumatic Stress Disorder.

* *p <*0.01; NA, Not applicable as both groups have the same values.

The distribution of the matched variables was identical between the OCD and control groups, with all standardized mean differences (SMDs) less than 0.001, reflecting perfectly matched samples.

Regarding the remaining psychiatric comorbidities, some differences were observed. PTSD was more prevalent in the OCD group (6.4%) compared to controls (4.8%), although the standardized mean difference (SMD = 0.071) indicated minimal imbalance. Similarly, schizoaffective disorder was slightly more common in the OCD group (6.5% vs. 5.7%, SMD = 0.035). Panic disorder and anxiety disorder were also more prevalent in the OCD group (6.2% vs. 3.1% for panic, SMD = 0.152; 58.6% vs. 54.1% for anxiety, SMD = 0.091), though these differences remained small. Depression diagnosis was higher in the OCD group (34.2% vs. 26.9%, SMD = 0.159). Overall, the exact matching process resulted in a highly balanced cohort, with SMD values below 0.2 for all variables, indicating that any differences between the groups were minimal and within acceptable thresholds for cohort studies.

### 3.3 Comparison of COVID-19 outcomes: OCD and control groups

**Figure 1:**
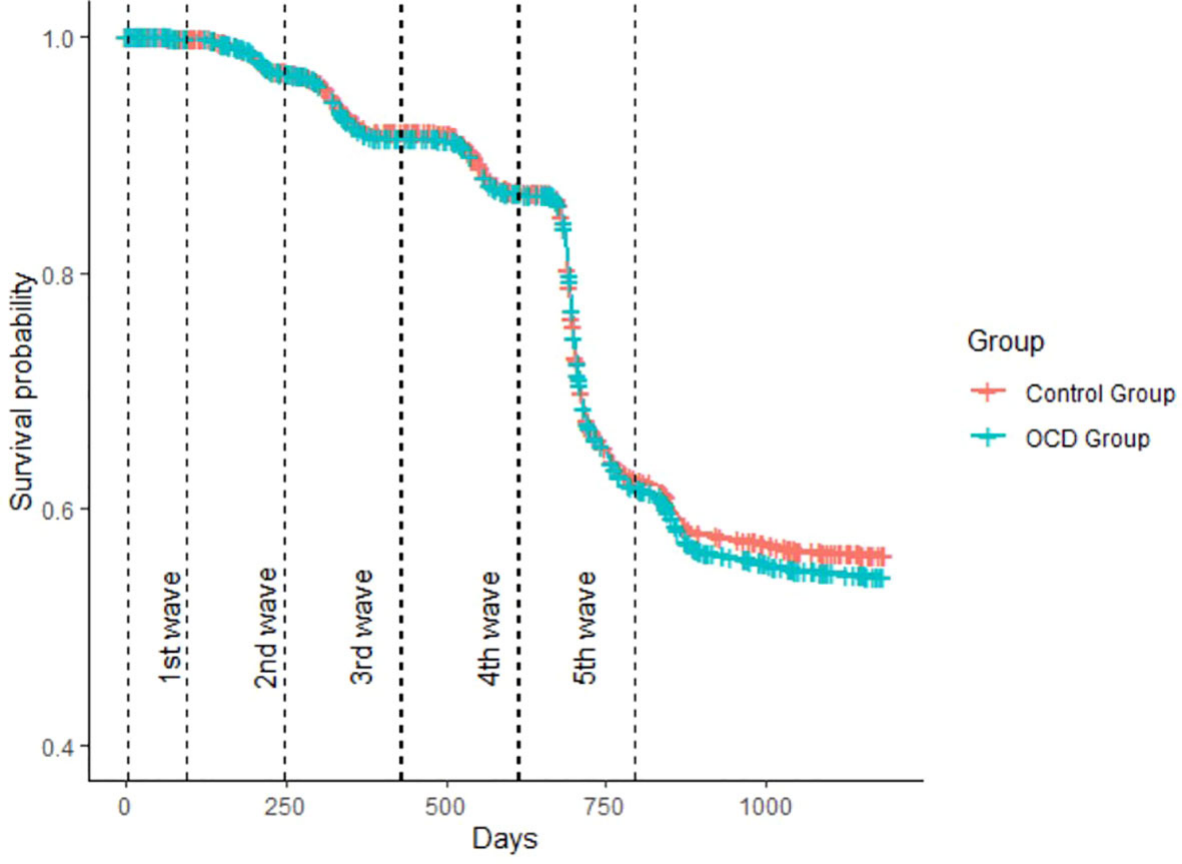
Kaplan-Meier Survival Analysis of Infection Timelines in OCD Patients vs. Matched Control Group. Kaplan–Meier plot showing days until the first positive COVID-19 test. Differences became significant in the time period after the fifth wave.

The results of the logistic regression analyses are presented in **Table 3**. Overall, the OCD group showed a slightly higher likelihood of testing positive for COVID-19 (45.1%) compared to the control group (43.3%), with the difference reaching statistical significance in both unadjusted (OR = 1.08 [1.03 - 1.13]) and adjusted models (OR = 1.10 [1.05 - 1.15]). Across the individual COVID-19 waves (first to fifth), there were no significant differences between the OCD and control groups in terms of positivity rates, as shown in the table. However, after the fifth wave, the OCD group had a significantly higher likelihood of testing positive (7.5%) compared to the control group (6.3%), with statistically significant results in both the unadjusted (OR = 1.20 [1.10 - 1.31]) and adjusted models (OR = 1.16 [1.06 - 1.27]) (see **Figure 1**). In terms of COVID-19 hospitalization, 0.6% of the OCD group and 0.5% of the control group were hospitalized, with no significant difference observed. For vaccination, there were no significant differences for the first or second doses between the two groups. However, the OCD group was more likely to receive the third vaccine dose (53.9% vs. 51.3%), with statistically significant differences in both the unadjusted (OR = 1.11 [1.06 - 1.16]) and adjusted models (OR = 1.05 [1.01 - 1.10]).

**Table 3:** Logistic regression results comparing OCD and control groups across COVID-19 outcomes.

Outcome	OCD Group, n (%)	Control Group, n (%)	χ^2^	*p-*value	OR [95% CI]	Adjusted OR [95% CI]
Positive COVID-19 Test	6935 (45.1%)	6647 (43.3%)	10.87	0.001*	1.08 [1.03 - 1.13]	1.10 [1.05 - 1.15]
First Wave Positive	12 (0.1%)	8 (0.1%)	0.45	0.502	1.50 [0.62 - 3.83]	1.55 [0.64 - 3.96]
Second Wave Positive	461 (3%)	444 (2.9%)	0.29	0.589	1.04 [0.91 - 1.19]	1.06 [0.93 - 1.21]
Third Wave Positive	839 (5.5%)	788 (5.1%)	1.62	0.202	1.07 [0.97 - 1.18]	1.08 [0.98 - 1.2]
Fourth Wave Positive	709 (4.6%)	748 (4.9%)	1.04	0.307	0.95 [0.85 - 1.05]	0.97 [0.87 - 1.07]
Fifth Wave Positive	3761 (24.5%)	3683 (24%)	1.05	0.305	1.03 [0.98 - 1.08]	1.05 [0.99 - 1.1]
Post-Waves Positive	1152 (7.5%)	973 (6.3%)	16.02	0.0001*	1.20 [1.10 - 1.31]	1.16 [1.06 - 1.27]
COVID-19 Hospitalizations	89 (0.6%)	71 (0.5%)	1.82	0.177	1.25 [0.92 - 1.72]	1.15 [0.84 - 1.58]
First Vaccine Dose	12046 (78.4%)	11981 (78%)	0.78	0.376	1.03 [0.97 - 1.08]	0.99 [0.94 - 1.04]
Second Vaccine Dose	11167 (72.7%)	11051 (71.9%)	2.15	0.142	1.04 [0.99 - 1.09]	1.00 [0.95 - 1.05]
Third Vaccine Dose	8284 (53.9%)	7879 (51.3%)	21.31	<0.0001*	1.11 [1.06 - 1.16]	1.05 [1.01 - 1.1]

Comparison of outcomes between OCD groups and controls group. *p <0.01.

OR, Odds Ratio. Adjusted OR, adjusted for psychiatric comorbidities that differed between the groups, specifically PTSD, schizoaffective disorder, panic disorder, anxiety, and depression.

### 3.4 Prediction of days to infection

Among those who tested positive for COVID-19, a regression analysis predicted the number of days until infection. This analysis, which included the group and various covariates, is detailed in **Table 4**. Being in the OCD group was significantly linked with a longer time until infection, averaging an additional 8.12 days compared to the control group, even when considering other covariates (b=8.12, SE=3.38, *p*=0.02).

**Table 4:** Regression analysis predicting days until COVID-19 infection among positive cases.

Variable	b [95% CI]	SE	t-statistic	*p*-value
OCD Group	8.12 [1.50, 14.75]	3.38	2.4	0.02*
Age at 2020	-1.35 [-1.55, -1.15]	0.1	-13.28	<0.001**
Male Gender	-9.46 [-16.20, -2.72]	3.44	-2.75	0.01*
SES: Low	-154.68 [-166.35, -143.01]	5.95	-25.98	<0.001**
SES: Medium	-61.68 [-70.12, -53.24]	4.31	-14.32	<0.001**
SES: No Data	-87.89 [-104.25, -71.53]	8.35	-10.53	<0.001**
ADHD Diagnosis	7.50 [-1.26, 16.27]	4.47	1.68	0.09
Bipolar Disorder Diagnosis	-7.71 [-25.46, 10.05]	10.05	-0.77	0.44
Panic Disorder Diagnosis	-5.83 [-20.10, 8.44]	8.27	-0.71	0.48
PTSD Diagnosis	-4.38 [-19.73, 10.98]	7.83	-0.56	0.58
Schizoaffective Disorder Diagnosis	17.75 [-0.94, 36.45]	9.54	1.86	0.06
Schizophrenia Diagnosis	-4.80 [-18.25, 8.66]	6.87	-0.7	0.48

Regression analysis predicting days until COVID-19 infection among positive cases. b represents the difference in the number of days until a positive test (a positive number indicates more days until infection, and a negative number indicates fewer days until infection). SES, Socioeconomic status compared to high; PTSD, Post-traumatic stress disorder; ADHD, Attention Deficit and Hyperactivity Disorder. *p < 0.05; **p <0.001.

## 4 Discussion

In the shadow of the COVID-19 pandemic, a global health crisis that has upended lives and reshaped societal norms, our study explored the intricate interplay between OCD and susceptibility to the virus. OCD, a condition that exists at the crossroads of mental health and behavioral reactions to perceived threats, becomes particularly pertinent in this scenario. Of particular importance is the OCD subtype marked by contamination fears, a symptom pattern that naturally aligns with the heightened hygiene protocols practices that have become crucial in combating COVID-19 (9, 21). While many studies examined how the COVID-19 pandemic influenced patients with OCD (11–13), to our knowledge, none examined whether OCD behaviors influenced the likelihood of contracting COVID-19.

For this research, we utilized the comprehensive database of Clalit Health Services, Israel’s largest healthcare provider. The study was driven by the hypothesis that OCD, especially its contamination-centric manifestations, may confer a protective effect against the novel coronavirus. This hypothesis is rooted in the evolutionary perspective, which posits that certain OCD obsessions and compulsions could be exaggerated forms of once-advantageous behaviors from the unprecedented focus on cleanliness and infection control that has marked the global response to the pandemic (8, 21). The results of our study challenge the hypothesis by suggesting that OCD does not offer protection against COVID-19. This surprising outcome questions the preconceived notions of both patients and researchers regarding the protective role of OCD behaviors against infectious diseases.

After extracting our sample of patients, we initiated our study with a meticulous matching process. This was necessary because the pre-matched group of individuals with OCD was notably younger, predominantly male, and was more frequently represented at both the lower and higher ends of the socioeconomic spectrum. There was also a marginally higher occurrence of comorbidities within this group, necessitating a careful adjustment to ensure a robust and unbiased comparison in our subsequent analysis.

We observed that the frequency of COVID-19 testing was similar between the matched control group and the OCD group. Despite this similarity, the OCD group had a marginally higher rate of positive COVID-19 tests. This difference only became significant after the fifth and final wave. This finding is particularly striking given that the OCD group was more likely to receive the vaccine booster (third dose) compared to the control group. The additional vaccination suggests that individuals with OCD may engage in behavior that is more cautious when it comes to vaccination, though this did not translate into lower infection rates.

Several factors might contribute to this unexpected pattern, contradicting our initial hypothesis. First, the variable of reporting behavior warrants consideration. Patients with OCD were possibly more likely to remain concerned about COVID-19 infection and continued to adhere to regular COVID-19 testing protocols even after the relaxation of pandemic-related restrictions. In contrast, the general population might have perceived a diminished necessity for testing in the presence of symptoms, leading to reduced reporting to healthcare providers. Unfortunately, our study was unable to ascertain whether the testing frequency for the OCD group differed before and after the end of the fifth wave, rendering this aspect inconclusive. Second, the behavior of OCD patients regarding safety measures may have impacted these results. Those with more rigorous adherence to safety protocols may have neglected other preventive behaviors, such as social distancing, due to mental fatigue, particularly post-vaccination (22). A detailed analysis of the time from vaccination to potential infection could have potentially supported this explanation. Third, the prolonged stress induced by the pandemic could have negatively affected patients’ immune responses, compromising their ability to combat infections effectively (23). Lastly, the increased comorbidity of ADHD in the OCD group, known as a risk factor for contracting COVID-19, may have contributed to the overall pattern (15, 18).

According to our data, individuals with OCD or anxiety diagnoses who were infected with COVID-19 experienced infection a few days later compared to those in the control group. On average, they were able to delay infection by 8 **±** 3.4 to 10 **±** 3.5 days. This represents a minimal difference over the timespan of a few years. This small delay may reflect a higher initial adherence to diligent preventive measures. However, over time, such behaviors may have become less effective, due to either behavioral fatigue or the unavoidable nature of certain transmission sources, such as close contact with household members (e.g., children) (24).

### 4.1 Limitations

Several limitations of our study warrant attention and are crucial for interpreting our findings.

First, the nature of our large-scale data analysis raises concerns about data quality and completeness, especially with the common issue of missing or inaccurately recorded information in medical and psychiatric databases. This can be especially problematic in the context of OCD, where diagnostic criteria and reporting standards may vary significantly across different healthcare settings. Additionally, some individuals may either remain undiagnosed or choose to seek private healthcare treatment to avoid their conditions from being recorded in public healthcare records. Notably, the prevalence of OCD observed in our study was 0.4%, which is significantly lower than the 2-8 times higher estimates commonly cited in the literature, suggesting underdiagnosis. However, this underreporting implies that the differences we observed between OCD patients and the general population might actually be understated, as the inclusion of undiagnosed OCD individuals in the non-OCD group could dilute the observed differences between groups. Conversely, our analysis did not distinguish between OCD subtypes, such as those with contamination fears, versus other forms not related to cleanliness, nor could we assess the severity of the disorder. This lack of specificity means that some individuals classified within the OCD group might not exhibit significant behavioral differences from the general population. As these issues potentially weaken our conclusion and strengthen our initial hypothesis, further individual-level studies are necessary to refine these insights.

Second, while the numbers and results of COVID-19 tests taken publicly are nationally transmitted and considered accurate, they do not include the frequency of at-home COVID-19 tests. The reported rate of positive tests also depends on individuals reporting their infections to community clinics, which may not always happen.

Third, the COVID-19 pandemic was characterized by extensive guidance from the Ministry of Health on minimizing the risk of infection, which the general population mostly adopted. This widespread adherence may have lessened the relative advantage of any natural protective behaviors associated with OCD, which might have been more beneficial before such guidance was available and government restrictions were implemented. This context is essential for understanding the potential impact of OCD behaviors on the risk of COVID-19 infection.

Finally, the positive test figures we derived indicate whether an individual was infected at any point; they do not reflect the frequency of infections per person. It is important to note that some individuals contracted the virus multiple times within the period under review, suggesting that a disparity in reinfection rates between the OCD and non-OCD populations might still be present.

### 4.2 Conclusion

Our findings contradict the hypothesis that a diagnosis of OCD provides protection against COVID-19. On the contrary, we observed a higher incidence of COVID-19 diagnoses among individuals with OCD. This outcome, coupled with several limitations and potential confounders, highlights a critical consideration: the most common hygiene behaviors associated with contamination-focused OCD, such as hand-washing, are primarily effective against diseases spread through direct contact, feco-oral routes or via contaminated surfaces, rather than respiratory illnesses like COVID-19. Therefore, while OCD-related hygiene practices may still offer some defense against various disease outbreaks, particularly those spread through non-respiratory means and were apparently ineffective in the COVID-19 pandemic. This finding is important for dispelling the misconception that intensified OCD-related hygiene practices could provide immunity against diseases like COVID-19. Understanding this can help correct false beliefs among OCD patients, thereby aiding in the provision of more accurate psychoeducation and enhancing the effectiveness of cognitive-behavioral therapy.

## Data Availability

The raw data supporting the conclusions of this article will be made available by the authors, without undue reservation.
